# Ion Channels and Transporters in Inflammation: Special Focus on TRP Channels and TRPC6

**DOI:** 10.3390/cells7070070

**Published:** 2018-07-04

**Authors:** Giuseppe A. Ramirez, Lavinia A. Coletto, Clara Sciorati, Enrica P. Bozzolo, Paolo Manunta, Patrizia Rovere-Querini, Angelo A. Manfredi

**Affiliations:** 1 Unit of Immunology, Rheumatology, Allergy and Rare Diseases, Università Vita-Salute San Raffaele, 20132 Milan, Italy; lavinia.coletto@gmail.com (L.A.C.); sciorati.clara@hsr.it (C.S.); bozzolo.enrica@hsr.it (E.P.B.); manunta.paolo@hsr.it (P.M.); rovere.patrizia@hsr.it (P.R.-Q.); manfredi.angelo@hsr.it (A.A.M.); 2 Unit of Immunology, Rheumatology, Allergy and Rare Diseases, IRCCS Ospedale San Raffaele, 20132 Milan, Italy; 3 Division of Immunology, Transplantation and Infectious Immunity, IRCCS Ospedale San Raffaele, 20132 Milan, Italy; 4 Unit of Nephrology, IRCCS Ospedale San Raffaele, 20132 Milan, Italy

**Keywords:** TRPC6, elementary immunology, inflammation, calcium, sodium, neutrophils, lymphocytes, endothelium, platelets

## Abstract

Allergy and autoimmune diseases are characterised by a multifactorial pathogenic background. Several genes involved in the control of innate and adaptive immunity have been associated with diseases and variably combine with each other as well as with environmental factors and epigenetic processes to shape the characteristics of individual manifestations. Systemic or local perturbations in salt/water balance and in ion exchanges between the intra- and extracellular spaces or among tissues play a role. In this field, usually referred to as elementary immunology, novel evidence has been recently acquired on the role of members of the transient potential receptor (TRP) channel family in several cellular mechanisms of potential significance for the pathophysiology of the immune response. TRP canonical channel 6 (TRPC6) is emerging as a functional element for the control of calcium currents in immune-committed cells and target tissues. In fact, TRPC6 influences leukocytes’ tasks such as transendothelial migration, chemotaxis, phagocytosis and cytokine release. TRPC6 also modulates the sensitivity of immune cells to apoptosis and influences tissue susceptibility to ischemia-reperfusion injury and excitotoxicity. Here, we provide a view of the interactions between ion exchanges and inflammation with a focus on the pathogenesis of immune-mediated diseases and potential future therapeutic implications.

## 1. Introduction

Ion exchanges between the intra- and extracellular spaces constitute fundamental mechanisms for the control of cell metabolism and activation state. Changes in the rate of crucial cell reactions such as energy accumulation, protein synthesis and cytoskeleton assembly in response to environmental stimuli are required for the long-term maintenance of homeostasis in complex organisms. Accordingly, genes encoding proteins expressed on the cell membrane to regulate its permeability to ions are crucial for the most complex intra- and intercellular tasks. In particular, ion channels (which account for up to 1% of the human genome [1] and allow the communication among different cells in an organism [1]. The nervous system is important to coordinate the ability of multicellular organisms to sense, adapt, record and possibly predict external stimuli [2]. The role of ion channels in neuronal activation has been investigated leading to seminal discoveries on their role in physiology and disease.

The current set of human ion channels genes marks the pillars of adaptive immunity [2], suggesting a link between ion channel specialisation and novel biological functions committed to host defence. Consistently, growing evidence is accumulating on the ability of ions, ion channels and transporters and their pharmacological modulators to influence the behaviour of the immune system at the cellular and clinical level, a phenomenon also known as elementary immunology [3].

Transient receptor potential (TRP) channels comprise a wide family of membrane proteins behaving as sodium/calcium permeable molecules. Their role in the deployment of the innate and adaptive immune response has received growing attention [4]. In this setting, TRP canonical channel 6 (TRPC6) has emerged as a modulator of calcium homeostasis in leukocytes and tissues involved by the inflammatory response. Here, we will review the potential mechanisms related to TRPC6 function considering its similarities and interactions with the elements of the cellular machinery committed to ion balance control.

## 2. Elementary Immunology: An Expanding Landscape

**Figure 1:**
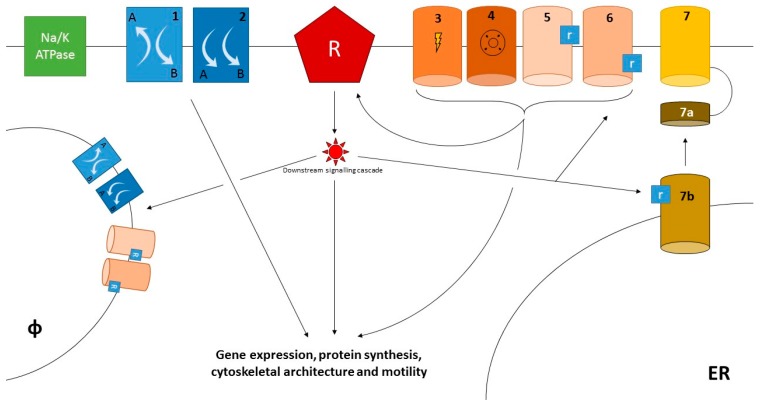
Ion channels and transporters. Ion channels and transporters may affect the behaviour of innate and adaptive immune cells at several levels. Under resting conditions, ion gradients between the intra- and extracellular space are actively generated through the Na/K ATPases. These gradients are exploited by transporters (1, 2) to trim the concentrations of other ions, including calcium. Cell activation after engagement of a cell-specific receptor (R), e.g., the BcR or TcR for lymphocytes or the FcR for myeloid cells, promotes the deployment of downstream signalling cascades that ultimately affect gene expression, protein synthesis and cause cytoskeletal remodelling, enabling cells to perform effector tasks such as chemotaxis, phagocytosis and release of antimicrobial moieties or cytokines. Activation of surface ion channels is integral to these events. A first set of ion channels are activated by physical or biochemical stimuli such as voltage (3), intracellular osmotic pressure (4) or engagement of extracellular (5) or intracellular (6) ligands, which in turn may be directly or indirectly induced by the activation of cell-specific receptors. Conversely, ion currents generated by voltage-operated or receptor-operated channels can exert feedback or feedforward effects on cell activating receptors. Specifically, raised calcium concentrations play a prominent role in mediating cell activation. However, to this regard store-operated calcium entry (SOCE, 7) generally provides a more significant contribution compared to voltage-operated or receptor-operated calcium entry (VOCE, ROCE). SOCE is propitiated by the activation of a inositol-1,4,5-triphosphate (IP_3_) receptor channel on the surface of the endoplasmic reticulum (ER, 7b). Increased intracellular IP_3_ concentrations are part of the changes induced by cell activation downstream cell-specific receptors (R). The release of calcium from ER stores is then sensed by adaptor proteins such as stromal interaction molecules (STIM; 7a), which in turn activate surface receptors (7), such as those of the ORAI family. Beside the cell surface, ion channels and transporters can also be expressed on intracellular compartments such as the phagolysosomes (φ). In this setting, they trim the endosomal pH, thus favouring the digestion of microbes and/or other dangerous moieties.

Ion channels and transporters affect immune responses [5] mainly by trimming endosomal pH [6,7,8,9] and intracellular calcium concentrations [3,10,11] (Table 1, Figure 1). This latter mechanism involves the intrinsic biophysical properties of a given ion channels or transporter and its ability to allow or facilitate the passage of calcium through the cell membrane. Changes in permeability of calcium channels or transporters can be triggered by either engagement of specific ligands (receptor-operated calcium entry, ROCE), feedforward responses to the release of calcium from intracellular stores (store-operated calcium entry, SOCE) and/or changes in cell polarisation (voltage-operated calcium entry, VOCE) and in the strength of the sodium driving force.

In the majority of cells, the most significant contribution to the rise of intracellular calcium concentrations is due to SOCE events [12,13,14,15], which are primed by the release of intracellular calcium stores downstream cell-specific activation pathways. These latter include the B- and T-cell receptor (BcR and TcR) or the Fc receptors pathways [15,16]. The main player in this setting is constituted by a functional triad comprising (a) an inositol-1,4,5-triphosphate (IP_3_) receptor channel expressed on the endoplasmic reticulum, which allows calcium to flow into the cytoplasm; (b) a set of cytoplasmic sensors called stromal interaction molecules (STIM); and (c) a membrane channel, bound to STIMs and composed of homo- or heteromers of members of the ORAI channel family [17,18]. The combination of ORAI and STIM protein is usually referred to as the calcium release-activated calcium channel (CRAC). The generation of IP_3_ is due to the activity of several types of phospholipases and is paired with the production of diacylglycerol (DAG), which in turn constitutes a ligand for several receptor/channels [19,20]. Intracellular phospholipases are involved in the signal cascades downstream BcR or TcR and can be modulated by the activity of ancillary ion-pathways such as those involving magnesium or zinc interchanges between the intra and extracellular space [21,22,23,24,25,26]. Auto- or paracrine adenosine triphosphate (ATP), adenosine diphosphate ribose (ADPR), and multiple other chemical ligands or physical stimuli modulate ROCE [27,28,29,30].

Voltage-gated calcium channels (Ca_v_) are required for leukocyte survival and are thought to be responsive to variations in cell polarisation [31]. Among the Ca_v_ subtypes, those belonging to the α1 pore-forming subunit family have been identified in lymphocytes [32]. Indirect pharmacological evidence suggests a role of Ca_v_ in myeloid-derived cells [31]. Sodium–calcium exchangers exploit gradients provided by the sodium-potassium ATPases to extrude calcium from the intracellular space. However, sodium depletion and prolonged cell depolarisation promote calcium entry through these transporters and favour cell activation [33,34].

Gain of function mutations in the sodium–calcium exchanger 1 (*NCX1*) gene, highly expressed at the level of arterial smooth muscle cells, which show a constitutionally slow recovery from depolarisation, associate with arterial hypertension, especially in the setting of sodium overload [35,36]. Enhanced activation and pro-inflammatory differentiation of macrophages and T-lymphocytes and enhanced formation of neutrophil extracellular traps occur in sodium-enriched extracellular environments [37,38,39,40,41,42]. *NCX1* risk alleles for salt-sensitive hypertension influences the course of nephritis in patients with systemic lupus erythematosus (SLE) [43]. While sodium overload can prompt NCX1 overactivity and enhanced cell activation, sodium-depleting conditions can also promote NCX1-mediated calcium responses and induce TNFalpha release from macrophages, mimicking lipopolysaccharide stimulation [44], and accelerate neutrophil recovery from an activation boost by increasing the speed of replenishment of intracellular calcium stores [11]. Voltage-gated potassium or sodium channels such as K_v_1.3 and Na_v_1.5, calcium-activated potassium channels such as K_Ca_3.1 and chloride channels, all play significant roles in the modulation of membrane polarisation, respectively, favouring or limiting calcium currents [27,45,46,47,48,49]. Macrophages from patients with cystic fibrosis, who have dysfunctional chloride currents due to mutations in the Cystic Fibrosis Transmembrane Conductance Regulator (*CFTR*) gene, are characterised by persistent pro-inflammatory activation and defective phagocytosis, facilitating chronic infection [50,51]. Ion channels and transporters also selectively exert a specifying modulatory role on geographically distinct compartments within immune-committed cells [52].

Besides the modulation of calcium currents, ion channels and transporters involved in the modulation of protons, sodium and calcium influence the functionality of immune cells by regulating the generation of reactive oxygen species (ROS) and interfering with the signalling pathways involved in the processing of immune stimuli [53,54]. Sodium-based transporters are fundamental for the modulation of energy uptake, which ultimately affect the cell lifespan [55]. Immune cells alternatively exploit ion channels and transporters to regulate the unconventional release of cytokines such IL-1β [29,56,57] or modulate their expression by modifying ion balances within the cell nucleus [58,59].

The variety of biochemical effects of ion channels and transporters on cell homeostasis ultimately influences the processing of immune stimuli [15]. Persistent alterations in the control of ion exchanges at the cellular level might ultimately contribute to hypersensitivity and autoimmunity while altered function of ion channels and transporters might influence the ability of target tissues to cope with inflammation-induced damage.

## 3. Multiple Roles for Members of the TRP Channel Family in Inflammation

TRP channels are widely expressed and contribute to the control of cell homeostasis. Thus, variations in the functionality of TRP might influence the physiological deployment of the immune response [4,123,124] (Table 1). Six subgroups within the TRP family have been described in humans according to structural homology between members: canonical (i.e., more similar to the original set of channels isolated in *Drosophila* [125], TRPC), vanilloid (TRPV), analogues of melastatin-1 receptor (TRPM), mucolipins (TRPML), polycystins (TRPP), endowed with ankyrin repeats (TRPA). The TRPN subclass owes its name to the NO-mechano-potential C receptor of the worm *Caenorhabditis elegans*. No members of this subclass have been identified in humans, with fishes being the only vertebrates in which this TRP subclass appears to be expressed [123,126].

TRPC channels play a major role in the modulation of calcium currents. In this setting, the formation of heteromeric complexes between different TRPC monomers might extend the spectrum of potential effects of this subclass of TRP channels on calcium homeostasis. In particular, TRPC1, has been proposed as a prototypic biochemical regulator for other membrane receptors thanks to its supposed ability to form heteromers [127,128,129,130]. TRPC1 might thus affect the activity of the ORAI/STIM complex as well as of other TRPC, such as TRPC6, to regulate SOCE. However, the evidence supporting this hypothesis is controversial due to the lack of highly specific anti-TRPC1 antibodies and to the need of tissue-restricted models of ORAI/STIM knockout (complete ORAI/STIM deficit is lethal at the embryonic stage in mice) [127]. TRPC1 is highly expressed in the endothelium, where it enhances vascular permeability after TNF/thrombin stimulation [65,66,67]. The potential ability of TRPC1 to orchestrate the function of other calcium channels is crucial for the maintenance of an intracellular calcium gradient for neutrophil chemotaxis in experimental models [52]. Animal models also suggest that TRPC1 plays a role in the control of IL1β release from macrophages [57]. Similarly, TRPC1 might affect the late effects of anaphylaxis by controlling TNF release from mast cells [121].

TRP channels play an even more relevant role as receptor-operated channels. TRPM2 and TRPC3 are expressed in a wide range of immune cells, including macrophages and lymphocytes, and play a role in T-cell activation after TcR engagement [69,70,76]. TRPM2 is responsible for a significant fraction of calcium currents within endothelial cells and neutrophils [71]. Accordingly, mice lacking TRPM2 show reduced neutrophil infiltrate and less extensive damage following myocardial infarction [72,73]. The main ligand of TRPM2 is ADPR, which lies downstream an intracellular stress-response pathway to ROS. ADPR-mediated activation of TRPM2, in turn, promotes the final step of a regulatory feedback loop that leads to the inhibition of NADPH-oxidase. This process is crucial in macrophages to control the extent of oxidative stress generation during the inflammatory response [54,74]. In this setting, lysosomal expression of TRPM2 is also required for phagocytosis [71,75]. In contrast to the anti-inflammatory effects of TRPM2 on macrophage activity, the role of TRPC3 on macrophage-driven inflammation is less clear. TRPC3 can be activated by DAG and is thought to contribute to vascular inflammation [77,78]. On the other hand, upregulation of TRPC3 downstream the pathway of brain-derived neurotrophic factor might have a protective role against neuronal inflammation and myocardial injury [28,91,92].

TRPV1 contributes to T cell activation by associating to TCR and responding to its engagement with increased calcium flux towards the intracellular space [95], whereas TRPV2 is upregulated by FCγR activation in macrophages and is involved in the deployment of phagocytosis and chemotaxis [96]. A recent study suggests that clustering of TRPV2 in lipid rafts is crucial for bacterial phagocytosis and is defective in patients with cystic fibrosis [51]. TRPM7 has also a crucial role in macrophage activation and is required for the physiological development of functional B- and T cells. Similar to the role of MagT1 receptor in T cells, TRPM7 responds to variation in Mg^2+^ concentrations (itself being more permeable to Mg^2+^ than to Ca^2+^) and enhances phospholipase activity downstream the BCR/TCR [98,99]. In addition, TRPM7 is crucial for mast cell survival and activation [100,101] as well as for macrophage survival and alternative activation [102]. TRPM7 might work by sensitising leukocytes to relatively low Mg^2+^ levels, rather than responding to acute variations in the concentration of the cation [103]. This is consistent with the evidence of lon8g-term, rather than sudden effects of TRPM7 deletion on leukocytes, with the partial compensatory role of exogenous Mg^2+^ [24] and with the clinical efficacy of MgSO_4_ in acute allergic reactions.

TRPC5, TRPV5 and TRPV6 have also been proposed to mediate calcium-dependent activation of leukocytes, although their precise pathways of activation have been less clearly defined [93,94,97,131,132,133]. TRPM4 exerts an inhibitory effect on calcium currents by promoting membrane depolarisation through calcium-induced sodium entry in macrophages and mast cells [105,106]. In addition, thanks to differential expression levels, TRPM4 exhibits distinct regulatory effects in Th1 and Th2 lymphocytes [107].

## 4. TRPC6 and Immune Responses

**Figure 2:**
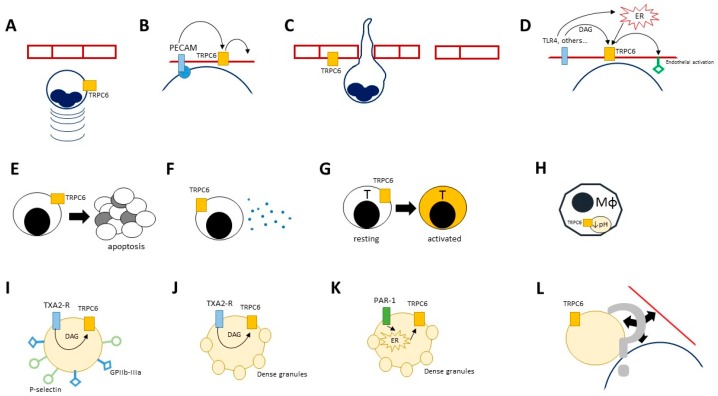
Effects of TRPC6 on immune cells. Activation of TRPC6 plays a critical role in the control of key cellular functions in several immune-committed cells, such as neutrophils (panel (**A**–**D**)), lymphocytes (panel (**E**–**G**)), macrophages (panel (**H**)), platelets (panel (**I**–**L**)) and the endothelium (panel (**A**–**D**,**L**)). TRPC6 contributes to neutrophil activation, adhesion to the vascular walls and extravasation by enhancing the stimulatory effects on chemo-attractants such as MIP-2 and CXCR2 (**A**); by promoting the downstream effects of endothelial cell adhesion molecules such as platelet/endothelial cell adhesion molecule (PECAM; (**B**)) or surface sensors of pro-inflammatory stimuli such as TLR-4 (**D**); by favouring the signal cascades that lead to looser transcellular junction between endothelial cells (**C**). Enhanced TRPC6 activation in lymphocytes might accelerate apoptosis, which could constitute a further trigger for inflammation in autoimmune disorders such as SLE (**E**). The expression of TRPC6 in T cells promotes cytokine release (**F**) and cell activation (**G**), which eventually translate in more aggressive inflammatory or allergic responses. In macrophages, TRPC6 is required for the acidification of endophagolysosomes (**H**). Platelets express high amounts of TRPC6 and might exploit its activation within ROCE (**I**,**J**) or SOCE (**K**) to undergo activation. Receptor-operated stimulation of TRPC6 downstream the thromboxane A2 (TXA2) pathway might be responsible for surface expression of crucial adhesion molecules such as GPIIb-IIIa or P-selectin (**J**) and for the release of platelet dense granules (**J**). This latter event might also occur as the result of TRPC6 activation after mobilisation of calcium from intracellular stores (**K**). Whether these events might impact on the interaction between platelets, leukocytes and the endothelium is still unknown (**L**).

TRPC6 is a member of a TRP subgroup with a probable dual role in SOCE and ROCE (Table 1) [20]. The fraction of calcium currents sustained by TRPC6 varies according to the inciting stimulus and to the cell type [134]. Evidence from neoplastic cell lines suggests that TRPC6-related calcium currents are crucial for the survival and activation of a multitude of histotypes [135,136,137,138,139,140]. The main physiological agonist of the receptor in the setting of ROCE is DAG. Conversely, endocytosis is the main mechanism for regulating TRPC6 function [141]. TRPC6 is expressed in a wide range of cell types, including neutrophils, lymphocytes, platelets and the endothelium (Table 1, Figure 2) [5]. During the acute phase response, TRPC6 plays a crucial role in neutrophil mobilisation as it enhances macrophage inflammatory protein 2 (MIP-2)- and CXCR2-related chemotactic responses by increasing Ca^2+^ concentration within the intracellular space and promoting actin-based cytoskeleton remodelling [79,80].

During trans-endothelial leukocyte migration TRPC6 acts on the endothelial side by mediating the downstream effects of platelet/endothelial cell adhesion molecule (PECAM/CD31) engagement, thus modulating endothelial permissibility [81]. TRPC6 contributes to loosen the endothelial junctions during acute inflammation, enhancing the effects of cellular and humoral immune mediators on target tissues [82,83]. Histamine-induced vascular leakage, which constitutes the core pathogenic mechanism in an acute hypersensitivity response, is also dependent on TRPC6, at least in animal models [142]. Finally, TRPC6 cooperates in lipopolysaccharide-induced endothelial activation after being itself activated by increasing intracellular concentrations of DAG, downstream the activation of Toll-like receptor 4 [143]. TRPC6 expressed on macrophage phagolysosomes is thought to promote their acidification and ultimately favour anti-microbial responses [8]. Chronically stimulated lung macrophages from patients with chronic obstructive pulmonary disease (COPD) express TRPC6 at high levels [144].

Calcium currents within T-lymphocytes are influenced by TRPC6 [145,146]. TRPC6 knockout dampens Th2-driven hypersensitivity responses in sensitised mice after airway allergen re-challenge [147] while sustained inward calcium currents due to TRPC6 may be indispensable for antimicrobial T cell responses during sepsis [148]. Notably, inhibitors of TRPC6 also have protective effects on the development of lymphocyte apoptosis [84]. This finding is in line with observations from others and us on the potential modulatory role of TRPC6 on cell death. TRPC6 influences endothelial apoptosis in an experimental model of atherosclerosis [148] and we observed that polymorphic gene variants of TRPC6 associate with susceptibility to apoptosis of peripheral blood mononuclear cells and diverging responses to the pharmacological inhibition of the channel in patients with SLE [84].

Enhanced apoptosis and unbalanced cell debris production to clearance ratios are fundamental, often calcium-dependent, events in autoimmunity, especially in the setting of SLE [149,150,151,152], a systemic autoimmune disease characterised by the production of autoantibodies against cell nuclear components and inflammatory manifestations involving multiple tissues and organs, such as skin and mucosal surfaces, joints, kidneys, serosae, central and peripheral nerves as well as circulating blood cells. TRPC6 gene variants might influence the secretory profile of SLE lymphocytes [84]. Retrospective clinical data from a well-characterised cohort of patients with SLE suggests the association between TRPC6 genetic polymorphisms and the risk of developing neuropsychiatric manifestations [85].

Megakaryocytes and platelets abundantly express TRPC6 on the plasma membrane [153]. TRPC6 promotes calcium entry after being activated by intracellular ligands such as DAG. TRPC6-mediated ROCE in human platelets is restricted to the thromboxane pathway and might induce the expression of surface molecules such as glycoproteins IIb-IIIa or P-selectin and the release of platelet-dense granules. TRPC6 might also be involved in dense granules secretion downstream the thrombin receptors pathway through SOCE [68]. These events play a role in haemostasis and accordingly, prolonged bleeding time and delayed formation of clots have been observed after TRPC6 inhibition or genetic deletion in mice [154,155]. However, evidence from murine models is controversial, as other authors reported normal platelet function and haemostasis in TRPC6 knockout mice [153]. Platelets are part of an interactive network that involves the endothelium and circulating leukocytes and sustains acute and long-term inflammatory responses [156,157]. While evidence has been provided to support a potential place for TRPC6 as a target for anticoagulation [154], little is known about the impact of TRPC6 inhibition in modulating platelet–leukocyte interactions and related clinical phenotypes [158,159,160,161].

## 5. Effects of TRPC6 Activation and Function on Inflamed Tissues

TRPC6 is a modulator of tissue susceptibility to inflammatory injuries. The channel is expressed in the lungs and is involved in the pathogenesis of ischaemia-reperfusion lung injury [162], septic acute lung injury [143] and idiopathic pulmonary arterial hypertension [163,164]. These events reflect the prominent expression and homeostatic action of TRPC6 on the lung vasculature, in particular at the level of the endothelium and of pulmonary artery smooth muscle cells [163,165]. TRPC6 might also play a role in the biology of other lung-residing cells [136]. Hypoxia-induced elevation of DAG vascular smooth muscle cells promotes ROCE through TRPC6 and subsequent vasoconstriction, which eventually exacerbates ischaemia [166]. Similar vasomotor effects have been demonstrated in aortic smooth muscle cells [167,168] and in the medial layer of coronary arteries in porcine models [169]. In addition, TRPC6-dependent surges in intracellular calcium concentrations contribute to the susceptibility of cardiomyocytes to ischaemia-reperfusion injury [170,171] and to long-term maladaptive responses leading to cardiac remodelling [172,173].

Animal models in which TRPC6 expression had been silenced revealed that TRPC6-mediated cellular responses prevented necroptosis of renal tubular epithelial cells [174], suggesting that TRPC6 contributes to protect the kidney from ischemia-reperfusion injury. Downregulation of TRPC6 influences the ability of mesangial cells to contract following angiotensin II stimulation [175,176] while overactive TRPC6 in podocytes promotes cytoskeletal remodelling due to sustained increased intracellular calcium concentrations with podosome disassembly and eventual proteinuria. Gain of function mutations of TRPC6 have been associated with familial forms of focal segmental glomerular sclerosis [177,178]. TRPC6 inhibition improves protein retention in rat models of nephrosis, suggesting that aberrant TRPC6 function might also exacerbate the clinical picture of patients with acquired forms of glomerular injury [179,180]. Accordingly, higher levels of TRPC6 RNA were found in urines of patients with more aggressive forms of lupus nephritis in a pilot study [181]. More recently, TRPC6 has also been implicated in the pathogenesis of tubular interstitial fibrosis [182].

These latter observations are consistent with the wider role of TRPC6 in sustaining wound healing and tissue remodelling responses after injury. In particular, in line with its role as a promoter of vascular smooth muscle cell contraction, TRPC6 is required for myofibroblast trans-differentiation from resting fibroblasts [183]. Recent evidence suggests the implication of this phenomenon in pulmonary fibrosis [184] and in intestinal strictures in patients with Crohn’s disease [185].

TRPC6 is expressed in neuronal tissues. TRPC6 activity in the nervous system seems to contrast the sequelae of brain ischaemia and reperfusion. Neurons are protected from post-ischaemic excitotoxicity by an indirect effect of TRPC6 on NMDA receptors [186,187] while under ischaemic conditions, TRPC6 degradation is enhanced in murine neurons by an IL17-dependent pathway. Inhibition of IL17 or of the downstream proteolytic enzyme calpain restores TRPC6 functions and reduces the area of post-ischemic necrosis [188]. A role for TRPC6 in modulating synaptic plasticity [189,190] and enhancing microglial activation [62] has been proposed.

## 6. Conclusions

The modulation of salt–water balance and electrolyte exchanges between the intra- and extra-cellular space has effects on the deployment of the immune response. Among the ion channels and transporters concurring to define the shape of the landscape of elementary immunology, TRPC6 seems to play a role in the regulation of several inflammatory events. More robust evidence from controlled human studies is required to pave the way to possible applications of TRPC6 as a target for diagnostic assessment or therapeutic intervention.

## Figures and Tables

**Table 1:** Functional impact of selected ion channels and transporters on inflammation.

**1. Modulation of Calcium Currents**					
**1.1 Through Direct Involvement in Calcium Influx/Efflux**					
**1.1.1 SOCE**					
*Channel*	*Permeability*	*Expression (immune cells)*	*Biological effects*	*Clinical correlates*	*Ref.*
ORAI1	Ca^2+^	Neutrophils, Lymphocytes	*Neutrophils*: proliferation, degranulation, cytokines production, cell polarization, migrational guidance with LFA1. *Lymphocytes*: B, T and NK cell proliferation, cytokine production and/or cytotoxicity in vitro; immunity to infection, T cell-mediated autoimmunity and inflammation, and allogeneic T cell responses in vivo; Treg cell development	CRAC channelopathywith immunodeficiency, autoimmunity, lymphoproliferation, muscular hypotonia and ectodermal dysplasia caused by mutations in STIM1 and ORAI1	[10,27,60,61]
ORAI2/3	Ca^2+^	Neutrophils, Lymphocytes	Cell proliferation, Cytokines production	ND	
STIM1	NA	Neutrophils, Lymphocytes, DC, mast cells	*Neutrophils:* phagocytosis and ROS production *Lymphocytes:* cytokine production in T and B cells, Treg functionality *Mast cells*: FcεR-triggered SOCE	ND	[13,14,27,62,63,64]
STIM2	NA			Mice deficient of STIM1/2 develop a lymphoproliferative disorder because of dysfunction of Treg cells.	
IP3Rs	Ca^2+^	All cells	Physiological development of B and T cells	ND	[16,17,18,19]
TRPC1	Ca^2+^, Na^+^	Endothelium	Enhanced vascular permeability after TNF/thrombin stimulation	ND	[65,66,67]
TRPC6	Ca^2+^, Na^+^	Platelets	Dense granules secretion after thrombin stimulation	ND	[68]
**1.1.2 ROCE**					
*Channel*	*Permeability*	*Expression (immune cells)*	*Biological effects*	*Clinical correlates*	*Ref.*
TRPM2	Ca^2+^, Na^+^	Neutrophils, lymphocytes, macrophages and DC	*Neutrophils*: increased activation and endothelial adhesion *Lymphocytes*: T cell proliferation and cytokine secretion *Macrophages and dendritic cells*: regulation of ROS formation	Mice lacking TRPM2 have milder ischaemia-reperfusion injury after myocardial infarction and attenuated experimental brain inflammation	[54,69,70,71,72,73,74,75]
TRPC3	Ca^2+^, Na^+^	Lymphocytes, macrophages	*Lymphocytes*: T cell activation downstream the TCR *Macrophages*: enhanced pro-inflammatory activation	Mice: accelerated atherosclerosis	[76,77,78]
TRPC6	Ca^2+^, Na^+^	Lymphocytes, neutrophils, endothelium, platelets	*Lymphocytes:* T cell activation *Neutrophils*: chemotaxis, *Endothelium*: enhanced endothelial permeability and activation *Platelets*: TXA2-dependent expression of glycoproteins IIb-IIIa and P-selectin, release of platelet dense granules	Mice: TRPC6 ko associates with milder airway hypersensitivity in asthma models Humans: single study suggesting an association between a TRPC6 polymorphism and neuropsychiatric SLE	[79,80,81,82,83,84,85]
TRPV4	Ca^2+^, Na^+^	Macrophages	Cell activation after lung barotrauma.	Mice: exacerbated lung inflammation in acute lung injury and increased inflammatory hyperalgesia	[30]
P2X_1_R, P2X_4_R	Ca^2+^, Na^+^	Lymphocytes, neutrophils, eosinophils, monocytes/macrophages, mast cells, and DC	*Lymphocytes*: T cell proliferation; cytokine production; thymocyte apoptosis *Macrophages*: PGE_2_ release, inflammasome activation	ND	[86,87]
P2X_7_R	Ca^2+^, Na^+^, other cations		*Lymphocytes*: T cell survival and cytokine production (downstream the TCR); T cell differentiation into Th17 vs. Treg *Macrophages*: activation of the NLRP3 inflammasome *Mast cells, eosinophils, DC*: inflammatory activation	Mice lacking P2X_7_R have attenuated allergic airway response, graft vs. host disease, allograft rejection	[88,89,90]
**1.1.3 VOCE**					
*Channel*	*Permeability*	*Expression (immune cells)*	*Biological effects*	*Clinical correlates*	*Ref.*
Ca_v_1.1-4	Ca^2+^	Lymphocytes	T cell survival, differentiation and progression to effector function	ND	[31,32]
**1.1.4 Direct calcium entry following upregulation**					
*Channel*	*Permeability*	*Expression (immune cells)*	*Biological effects*	*Clinical correlates*	*Ref.*
TRPC3	Ca^2+^, Na^+^	Macrophages/microglia	Regulation of cellular activation	Mice: reduced brain inflammation and post-ischaemic myocardial damage	[28,91,92]
TRPC5	Ca^2+^, Na^+^	Lymphocytes	Inhibition of Teff activation by Treg	Mice: protection from experimental arthritis	[93,94]
TRPV1	Ca^2+^, Na^+^	T lymphocytes	Cell activation (by associating to TCR)	ND	[95]
TRPV2	Ca^2+^, Na^+^	Macrophages	Phagocytosis, chemotaxis, following FCγR activation	Mice: TRPV2 deletion prompts accelerated mortality in bacterial infections Humans: cystic Fibrosis macrophages exhibit a defect in TRPV2-mediated calcium influx	[51,96]
TRPV5,6	Ca^2+^, Na^+^	Lymphocytes	Cell activation and proliferation (the channels are constitutively active and regulated by endocytosis or at gene expression level).	ND	[97]
**1.2 Through intracellular second messengers**					
*Channel*	*Permeability*	*Expression (immune cells)*	*Biological effects*	*Clinical correlates*	*Ref.*
TRPM7	Mg^2+^, Ca^2+^	Lymphocytes, macrophages, mast cells	Lymphocytes: activation downstream BCR and TCR; thymocyte development; production of thymocyte growth factor Macrophages: survival and M2 polarisation Mast cells: survival and activation	ND	[98,99,100,101,102,103]
MAGT1	Mg^2+^	Lymphocytes	CD4+ T cell development and activation; immunity to EBV	XMEN syndrome (X-linked mutations in MAGT1)	[104]
ZIP6	Zn^2+^	T cells, DC	*T cells*: sustained calcium currents enhancing TCR-related pathways and promoting T cell activation DC: inhibition of maturation for antigen presentation	Genetically determined zinc deficit (mutated ZIP4 in the intestinal mucosa) causes acrodermatitis enteropathica with immunodeficiency	[26]
ZIP8		T cells	Sustained calcium currents enhancing TCR-related pathways and promoting T cell activation		
**1.3 Through alterations of cell polarisation**					
*Channel*	*Permeability*	*Expression (immune cells)*	*Biological effects*	*Clinical correlates*	*Ref.*
NCX1	Ca^2+^, Na^+^	NeutrophilsMacrophages	Neutrophils: recovery from activation Macrophages: activation, cytokine (TNF) secretion	A single association study suggests potential links among NCX polymorphisms and SLE phenotypes (including severe nephritis)	[11,43,44]
NKCC2	Na^+^, K^+^, 2Cl^−^	Lymphocytes	Adaptation to extracellular hypertonicity, which eventually leads to the activation of the p38/MAPK → NFAT5 → SGK pathway, which favours Th17 differentiation	ND	[37]
ENaC	Na^+^				
NHE1	Na^+^, H^+^				
TRPM4	Na^+^, Ca^2+^	Lymphocytes, macrophages and DC, mast cells	*Lymphocytes:* T helper motility and cytokine production (IL2, IL4, and IFNγ). *Macrophages*: phagocytosis and cytokine release *DC*: motility *Mast cells*: regulation of cell activation	Mice: lack of TRPM4 associates with reduced survival in sepsis and more intense anaphylaxis	[105,106,107,108]
GABA_A_-R	Cl^−^	Lymphocytes, macrophages and DC, neutrophils	Inhibition of cell activation	In preclinical models GABAergic drugs, protects against type 1 diabetes (T1D), experimental autoimmune encephalomyelitis (EAE), collagen-induced arthritis (CIA), contact dermatitis and allergic asthma. Treatment with gabapentin and pregabalin improved psoriasis (case report).	[49]
CFTR	Cl^−^	Lymphocytes, macrophages	*Lymphocytes*: modulation of cytokine secretory profile (IL5, IL10) in T cells *Macrophages*: cytokine release, phagocytosis	Cystic fibrosis	[51,109]
K_v_1.3	K^+^	Lymphocytes	Enhanced activation of the NLRP3 inflammasome and of IL1β production. Enhanced cell survival and prolonged activation.	A single phase Ib study on dalazatide (a specific K_v_1.3 inhibitor) shows promise. Applications in SLE have been proposed.	[110,111,112]
KCa3.1	K^+^	Lymphocytes, macrophages, endothelium	*Lymphocytes*: sustained TCR-induced calcium currents to support long-lasting effector functions. *Macrophages*: activation, chemotaxis, infiltration of atherosclerotic plaques *Endothelium*: proliferation	Encouraging evidence of efficacy of K_Ca_3.1blockers in several models of inflammatory vasculopathy and autoimmunity.	[47,113,114,115,116]
Na_v_1.5 (SCN5A)	Na^+^	T cells	Positive selection of thymocytes	ND	[46]
P2X_7_R	Ca^2+^, Na^+^ and other cations	Macrophages	Cell death for prolonged depolarisation in case of sustained receptor ligation.	ND	[117]
**1.4 Through alterations in the geographical distribution of intracellular calcium**					
*Channel*	*Permeability*	*Expression (immune cells)*	*Biological effects*	*Clinical correlates*	*Ref.*
TRPC1	Ca^2+^, Na^+^	Neutrophils	Cell polarisation for chemotaxis	ND	[65,66,67]
**2. Modulation of intracellular pH and production of reactive oxygen species**					
*Channel*	*Permeability*	*Expression (immune cells)*	*Biological effects*	*Clinical correlates*	*Ref.*
TRPM2	Ca^2+^, Na^+^	Macrophages and DC	*Macrophages and DC*: regulation of ROS formation, phagocytosis and bacterial killing	ND	[71,75]
H_v_1/VSOP	H^+^	lymphocytes, granulocytes, macrophages and DC	All cells: phagocytosis and ROS production B cells: BCR signalling	Mice: loss of the receptor prompts impaired killing of phagocytosed bacteria, ROS production and migration by leukocytes and impaired antibody responses.	[15,53]
NCX	Ca^2+^, Na^+^	DC	Activation of NADPH oxidase and polarisation towards pro-inflammatory DC.		[42]
ENac	Na^+^				
NHE	Na^+^, H^+^				
**3. Modulation of endosomal pH**					
*Channel*	*Permeability*	*Expression (immune cells)*	*Biological effects*	*Clinical correlates*	*Ref.*
TRPC6	Ca^2+^, Na^+^	Macrophages	Phagocytosis and bacterial killing	ND	[8]
TRPM2	Ca^2+^, Na^+^	Macrophages and DC	Phagocytosis and bacterial killing	ND	[71,75]
Proton ATPases	H^+^	Macrophages	Phagocytosis and bacterial killing	ND	[118]
Na_v_1.5 (SCN5A)	Na^+^	Macrophages	endosomal acidification and phagocytosis. Possible polarisation towards an antinflammatory phenotype	Mice: enhanced recovery from EAE.	[45,119]
CLIC 1	Cl^−^	Macrophages and DC	Phagocytosis, antigen processing and presentation.	ND	[9,120]
**4. Modulation of other intracellular signalling pathways**					
*Channel*	*Permeability*	*Expression (immune cells)*	*Biological effects*	*Clinical correlates*	*Ref.*
TRPC1	Ca^2+^, Na^+^	Macrophages, Mast cells	*Macrophages*: inhibition of IL1β through other ion channels and transporters *Mast-cells*: inhibition of calcium-dependent release of TNF in the late phase of cell activation	Mice: delayed recovery from anaphylaxis	[77,121]
**5. Other effects**					
*Channel*	*Permeability*	*Expression (immune cells)*	*Biological effects*	*Clinical correlates*	*Ref.*
SLC5A11	Na^+^, glucose	Leukocytes (low)	Leukocytes: control of cell osmolarity under hypernatriemic environment, energy uptake, TNF-dependent apoptosis	Polymorphisms associated with susceptibility to SLE	[55,122]
CLIC 1	Cl^−^	Macrophages	Modulation of cytokine gene expression and processing (conflicting results)	ND	[9,58,59]
CLIC 4	Cl^−^				

Abbreviations. Ca_v_: voltage-gated calcium channels; CFTR: cystic fibrosis transmembrane conductance regulator; CLIC: chloride intracellular channels; DC: dendritic cells; EAE: experimental allergic encephalomyelitis; ENaC: epithelial sodium channel; GABA_A_-R: gamma-aminobutyric acid receptor type A; NADPH: nicotinamide adenine dinucleotide phosphate; NCX1: sodium-calcium exchanger 1; ND: not determined; NHE1: sodium-hydrogen exchanger 1; NKCC2: sodium-potassium-2 chloride exchanger; PGE2: prostaglandin E2; ROCE: receptor-operated calcium entry; SLC5A11: sodium glucose cotransporter; SOCE: store-operated calcium entry; STIM: stromal interaction molecule; TCR, T cell receptor; TRP: transient receptor potential channel; TXA2: thromboxane A2; VOCE: voltage-operated calcium entry; VSOP: voltage-sensing domain only protein; XMEN, X-linked immunodeficiency with Mg^2+^ defect and EBV infection and neoplasia; ZIP: zinc-regulated transporter (ZRT)/iron regulated transporter(IRT)-like protein.
